# Tuberculosis incidence is high in HIV-infected African children but is reduced by co-trimoxazole and time on antiretroviral therapy

**DOI:** 10.1186/s12916-016-0593-7

**Published:** 2016-03-23

**Authors:** Angela M. Crook, Anna Turkova, Victor Musiime, Mutsa Bwakura-Dangarembizi, Sabrina Bakeera-Kitaka, Patricia Nahirya-Ntege, Margaret Thomason, Peter Mugyenyi, Philippa Musoke, Adeodata Kekitiinwa, Paula Munderi, Kusum Nathoo, Andrew J. Prendergast, A. Sarah Walker, Diana M. Gibb, And The ARROW Trial Team

**Affiliations:** MRC Clinical Trials Unit at UCL, London, UK; Joint Clinical Research Centre, Kampala, Uganda; Makerere University College of Health Sciences, Kampala, Uganda; Department of Paediatrics and Child Health, University of Zimbabwe Medical School, Harare, Zimbabwe; Baylor College of Medicine Children’s Foundation, Kampala, Uganda; MRC/UVRI Uganda Research Unit on AIDS, Entebbe, Uganda; MU-JHU Care Ltd, Kampala, Uganda; Blizard Institute, Queen Mary University of London, London, UK

**Keywords:** Antiretroviral therapy, HIV, Incident tuberculosis, Pediatric

## Abstract

**Background:**

There are few data on tuberculosis (TB) incidence in HIV-infected children on antiretroviral therapy (ART). Observational studies suggest co-trimoxazole prophylaxis may prevent TB, but there are no randomized data supporting this. The ARROW trial, which enrolled HIV-infected children initiating ART in Uganda and Zimbabwe and included randomized cessation of co-trimoxazole prophylaxis, provided an opportunity to estimate the incidence of TB over time, to explore potential risk factors for TB, and to evaluate the effect of stopping co-trimoxazole prophylaxis.

**Methods:**

Of 1,206 children enrolled in ARROW, there were 969 children with no previous TB history. After 96 weeks on ART, children older than 3 years were randomized to stop or continue co-trimoxazole prophylaxis; 622 were eligible and included in the co-trimoxazole analysis. Endpoints, including TB, were adjudicated blind to randomization by an independent endpoint review committee (ERC). Crude incidence rates of TB were estimated and potential risk factors, including age, sex, center, CD4, weight, height, and initial ART strategy, were explored in multivariable Cox proportional hazards models.

**Results:**

After a median of 4 years follow-up (3,632 child-years), 69 children had an ERC-confirmed TB diagnosis. The overall TB incidence was 1.9/100 child-years (95 % CI, 1.5–2.4), and was highest in the first 12 weeks following ART initiation (8.8/100 child-years (5.2–13.4) versus 1.2/100 child-years (0.8–1.6) after 52 weeks). A higher TB risk was independently associated with younger age (<3 years), female sex, lower pre-ART weight-for-age Z-score, and current CD4 percent; fewer TB diagnoses were observed in children on maintenance triple nucleoside reverse transcriptase inhibitor (NRTI) ART compared to standard non-NRTI + 2NRTI.

Over the median 2 years of follow-up, there were 20 ERC-adjudicated TB cases among 622 children in the co-trimoxazole analysis: 5 in the continue arm and 15 in the stop arm (hazard ratio (stop: continue) = 3.0 (95 % CI, 1.1–8.3), *P* = 0.028). TB risk was also independently associated with lower current CD4 percent (*P* <0.001).

**Conclusions:**

TB incidence varies over time following ART initiation, and is particularly high during the first 3 months post-ART, reinforcing the importance of TB screening prior to starting ART and use of isoniazid preventive therapy once active TB is excluded. HIV-infected children continuing co-trimoxazole prophylaxis after 96 weeks of ART were diagnosed with TB less frequently, highlighting a potentially important role of co-trimoxazole in preventing TB.

**Electronic supplementary material:**

The online version of this article (doi:10.1186/s12916-016-0593-7) contains supplementary material, which is available to authorized users.

## Background

Tuberculosis (TB) remains a leading cause of morbidity and mortality among HIV-infected patients in low-income countries [1]. The HIV epidemic has shifted the peak age of TB in adults to younger ages, resulting in higher TB exposure and TB burden in children [2].

Antiretroviral therapy (ART) has been shown to reduce TB incidence in adults and children [3–8]. Two recent randomized controlled trials in adults showed that early compared to deferred ART initiation reduced TB in individuals with high CD4 counts [9, 10]. However, there remains an elevated risk of TB compared to HIV-uninfected individuals [11]. Despite improved access to ART, TB remains a serious co-infection in HIV-infected children, accounting for nearly 30 % of deaths in this population, with the highest burden in sub-Saharan Africa [1, 12].

Recently, the role of co-trimoxazole in TB prevention has been investigated. Observational evidence suggests that extended use of this inexpensive and widely available prophylactic antibiotic may reduce the risk of developing TB in adults [13]. This is supported by in vitro data suggesting that co-trimoxazole has anti-mycobacterial activity [14] and synergistic activity with rifampicin [15]. However, there are no randomized data on the role of co-trimoxazole in TB prevention.

The ARROW trial followed 1,206 HIV-infected children in Uganda and Zimbabwe for up to 5 years after ART initiation and included a randomized intervention to stop or continue co-trimoxazole after 96 weeks on ART [4]. The trial showed that that extended use of co-trimoxazole after 2 years of ART reduced hospitalizations for malaria and non-malaria infections, such as pneumonia and sepsis [16]. Long-term co-trimoxazole has subsequently been recommended for all HIV-infected children [17].

Using data from the ARROW trial, the present study had two aims: first, to calculate the incidence of TB from ART initiation over a median of 4 years follow-up on ART; and, second, to compare TB diagnoses in children randomized to stop or continue co-trimoxazole after 96 weeks on ART.

## Methods

### ARROW trial

The ARROW trial design, methods, and results have been described elsewhere [16, 18]. Briefly, ARROW was a randomized controlled factorial trial comparing outcomes of children randomized to either clinically- or laboratory-driven monitoring and (simultaneously in a factorial design) to either a standard three-drug or an induction-maintenance first-line ART regimen. Children were randomized to one of three arms: Arm A (lamivudine/abacavir/non-nucleoside reverse transcriptase inhibitor (NNRTI) throughout); Arm B (lamivudine/abacavir/zidovudine/NNRTI until week 36, with subsequent lamivudine/abacavir/NNRTI); or Arm C (lamivudine/abacavir/zidovudine/NNRTI until week 36, with subsequent lamivudine/abacavir/zidovudine). A total of 1,206 children and adolescents, median age 6 years (range, 3 months to 17 years), eligible for ART following WHO 2006 [19] guidelines, were enrolled between March 2007 and November 2008 from three centres from Uganda and one in Zimbabwe.

All children started once daily co-trimoxazole at enrolment if they were not already taking it, as per the WHO 2006 [19] guidelines (200 mg sulfamethoxazole and 40 mg of trimethoprim, 400 mg sulfamethoxazole and 80 mg of trimethoprim, or 800 mg of sulfamethoxazole and 160 mg of trimethoprim for a body weight of 5–15, 15–30, or >30 kg, respectively). Isoniazid preventative therapy was not routinely used, and TB screening pre-enrolment was based on standard elicitation of symptoms. After enrolment, children were followed at weeks 4, 8, and 12, and then 12 weekly for up to 5 years. Children saw a nurse at every visit who solicited specific symptoms and referred the child to the doctor if unwell. Height and weight were measured at every visit and converted to Z-scores using UK reference standards [20]. Children saw a doctor routinely every 12 weeks for assessments including a full blood count and CD4 T-cell count. Only 3 % of children were lost to follow-up; 95 % remained on first-line ART over a median of 4 years and 96 % were alive at the end of the trial. The trial included an additional (open-label) randomization 96 weeks after ART initiation to either stop or continue taking co-trimoxazole prophylaxis. Children over 3 years of age who had been receiving ART for at least 96 weeks and were using insecticide-treated bed-nets (in malaria-endemic areas) were eligible for this randomization. Children with previous *P. jirovecii* pneumonia were excluded. For the combined primary endpoint of death and hospitalizations, the trial showed that prolonged use of co-trimoxazole was beneficial [16].

All caregivers provided written informed consent to participate in the study and for future publication of data: children and adolescents provided consent or assent, depending on age and knowledge of HIV status. The trial was approved by ethics committees in Uganda (Joint Clinical Research Centre IRB Office), Zimbabwe (Medical Research Council of Zimbabwe), and the UK (UCL Research Ethics Committee).

### Diagnosis of TB in ARROW

Data on TB diagnosis were collected on a standardized form, from enrolment and throughout follow-up. TB was reported as either definitive or presumptive (as per WHO diagnostic criteria for HIV-infected children) [21]. A diagnosis of TB was made based on suggestive clinical features with available supportive investigations, including the tuberculin skin test, chest X-ray or other imaging, and sputum microscopy with or without culture. TB was categorized by site of infection as pulmonary, disseminated extrapulmonary, and tuberculous lymph node disease.

All TB diagnoses and causes of death were adjudicated blind to randomized arm by an independent Endpoint Review Committee (ERC) using clinical summaries of the event provided in real-time by clinicians managing the children, and all routine and non-routine laboratory data including history of TB contact, clinical presentation, microbiological and radiological investigations, and response to TB treatment. Deaths were classified as TB-related when TB was adjudicated as one of the causes of death. The ERC also adjudicated whether, in their opinion, the TB event was likely due to immune reconstitution inflammatory syndrome (IRIS); there are no validated IRIS definitions in children.

In order to exclude the possibility of relapses from previous TB infection, children with a reported past history of TB (including prevalent TB at enrolment) were excluded from the analyses.

### Statistical methods

#### TB incidence analysis

Time to TB diagnosis was defined as time from ART initiation to the date of the first TB diagnosis. The incidence of TB per 100 child-years was calculated as the number of confirmed TB diagnoses occurring within each of the following time periods divided by the total accrued child-time for that same period: (1) 0–12 weeks; (2) 12–52 weeks; (3) >52 weeks. A smoothed estimate of the incidence over time was also estimated from a flexible parametric model [22]. A multivariable Cox proportional hazards model was used to explore potential risk factors for TB, including baseline age, sex, center, initial randomized ART strategy (Arm A, B, C, as above), randomized monitoring strategy (clinically- vs. laboratory-driven monitoring), WHO clinical stage 3/4, and baseline and time-updated weight/height-for-age Z-scores and CD4 (count and %), including all factors with *P* <0.1 in a univariable model in the final adjusted model. We did not include viral load as this was measured retrospectively in selected samples in a subset of children post-baseline.

### Effect of stopping co-trimoxazole

For the co-trimoxazole analysis, time zero was the date of the co-trimoxazole randomization, which occurred more than 96 weeks after ART initiation. Time to TB diagnosis in this analysis was defined as the time from co-trimoxazole randomization to diagnosis of TB, including any recurrences in children who had had a previously confirmed TB diagnosis during follow-up, but excluding children with prevalent or prior TB at ART initiation (since these diagnoses could not be confirmed). Kaplan–Meier and multivariable Cox proportional hazards models were fitted to estimate the unadjusted and adjusted effect of stopping compared to continuing co-trimoxazole on the risk of TB diagnosis. Covariates considered for a multivariable adjusted model were as for the TB incidence analysis, measured at the time of co-trimoxazole randomization.

All analyses were performed in Stata (version 13).

## Results

### TB incidence analysis

**Fig. 1:**
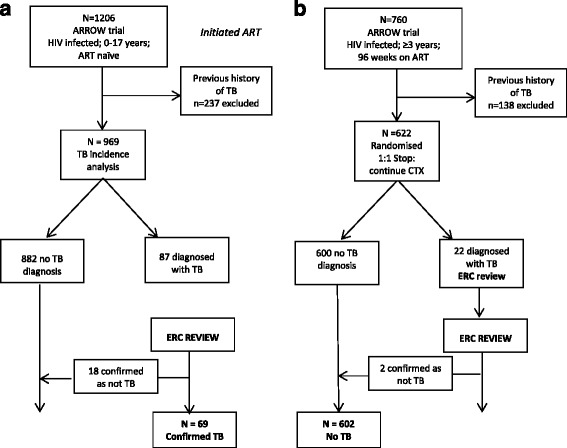
Analysis populations: (**a**) main tuberculosis incidence analysis and (**b**) co-trimoxazole analysis

Of the 1,206 children in the ARROW trial, 237 had a history of TB and were excluded, leaving 969 in the analysis (Fig. 1a). Over a median of 4 years follow-up from ART initiation, 87 children (9 %) were diagnosed with TB; 18 TB diagnoses (14 pulmonary and 4 extra-pulmonary) were adjudicated as not TB by the ERC based on established alternative diagnoses, leaving 69 incident TB cases (61 pulmonary, 3 extra-pulmonary, and 5 lymph node TB) and 900 children with no TB at the end of follow-up (Fig. 1a). A total of 55 (80 %) of the 69 TB cases adjudicated by the ERC were presumptive diagnoses; 14 (20 %) were definitive.

**Fig. 2:**
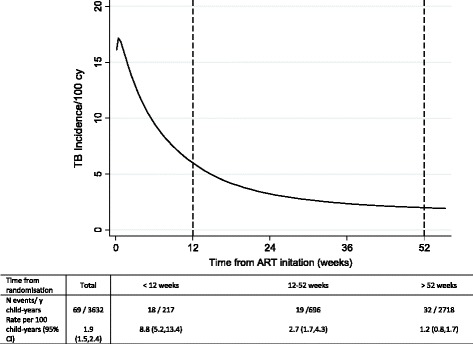
Tuberculosis incidence over time after antiretroviral therapy initiation

Based on the 69 ERC-adjudicated TB cases, the overall incidence of TB was 1.9 per 100 child-years (95 % CI, 1.5–2.4), which varied over time: 8.8/100 child-years for the first 12 weeks, 2.7/100 child-years between 12 and 52 weeks, and 1.2/100 child-years thereafter (Fig. 2). The smoothed hazard function indicates a peak in the first few weeks following ART initiation with the risk falling rapidly thereafter.

Table 1 shows the characteristics of children at ART initiation. Overall, the median age was 5 years (IQR, 2–9), 488 (50 %) were male, and 712 (73 %) were recruited from sites in Uganda and 257 (27 %) from Zimbabwe. Children with an ERC-adjudicated incident TB diagnosis were marginally more likely to be aged <3 years at ART initiation (45 % vs. 34 % without TB, *P* = 0.07), and to be female (59 % vs. 49 %, *P* = 0.09); they were significantly more likely to have a lower CD4 percent (median 8 % vs. 13 % for those without TB, *P* <0.001), lower height- and weight-for-age Z scores (median −3.0 and −2.8, respectively, vs. −2.3, and −1.8, respectively, both *P* <0.001); and to already have WHO stage 3 or 4 HIV disease (75 % vs. 62 %, *P* = 0.03) at ART initiation. Additional file 1 shows the same characteristics including children (*n* = 237) with a history of TB, indicating broadly similar results. Of note, a history of TB was not found to be associated with future TB in this cohort.

**Table 1:** Baseline characteristics of children by subsequent tuberculosis (TB) status

	TB	No TB	Total	*P* value
	69 (7 %)	900 (93 %)	969	
Age (at baseline)				
Median, IQR (years)	5 (2–11)	5 (2–9)		
< 3 years	31 (9)	307 (91)	338	0.07
Sex				
Male	28 (6)	460 (94)	488	0.09
Female	41 (9)	440 (91)	481	
Centre				
Entebbe	16 (9)	163 (91)	179	0.35
JCRC	16 (6)	248 (94)	264	
Harare	14 (6)	243 (94)	257	
PIDC	23 (9)	246 (91)	269	
Height (median IQR)				
Height-for-age Z score	−3.0 (−4.0 to –1.8)	−2.3 (−3.3 to –1.3)		<0.001
Weight (median IQR)				
Weight-for-age Z score	−2.8 (−4.1 to –1.8)	−1.8 (−2.8 to –0.9)		<0.001
WHO stage				
3 or 4	52 (8)	561 (92)	613	0.03
CD4 (median, IQR)				
CD4 %	8 (5–13)	13 (7–19)		0.001
CD4 count^a^	136 (33–250)	253 (93–405)		0.07
Initial ART				
3TC ABC EFV	6 (6)	99 (94)	105	0.03
3TC ABC NVP	24 (11)	190 (89)	214	
ZDV 3TC ABC EFV	9 (4)	217 (96)	226	
ZDV 3TC ABC NVP	30 (7)	394 (93)	30	
Randomization				
A (3TC/ABC/NNRTI throughout)	30 (9)	289 (91)	319	
B (3TC/ABC/NNRTI throughout, ZDV until week 36)	24 (7)	301 (93)	325	
C (3TC/ABC/ZDV throughout, NNRTI until week 36)	15 (5)	310 (95)	325	0.06
Randomization				
Clinical monitoring	35 (7)	449 (93)	484	
Laboratory monitoring	34 (7)	451 (93)	485	0.89

Values are n (row %) unless otherwise stated

^a^In those over 5 years

*3TC* Lamivudine, *ABC* Abacavir, *EFV* Efavirenz, *IQR* Interquartile range, *JCRC* Joint Clinical Research Centre, *NNRTI* Non-nucleoside reverse transcriptase inhibitor, *NVP* Nevirapine, *PIDC* Paediatric Infectious Diseases Clinic, *ZDV* Zidovudine

In univariable analysis, a 3- or 4-drug ART regimen that included nevirapine (compared to efavirenz) was associated with an increased risk of TB (*P* = 0.03). A lower proportion of children in Arm C (initial 4-drug regimen decreased to 3-drug NRTI-only regimen after 36 weeks) developed TB compared to other groups (*P* = 0.06). There was no difference in TB incidence between patients randomized to either clinical- or laboratory-driven monitoring.

Table 2 shows unadjusted and adjusted hazard ratios from the multivariable Cox model, including effects of the time-updated covariates. The following factors were independently associated with the risk of developing TB: younger age at ART initiation (*P* = 0.04), female sex (*P* = 0.01), lower current weight-for-age (*P* = 0.001), and lower current CD4 percent (*P* <0.001). Current height-for-age and pre-ART WHO stage did not independently predict TB risk. Baseline weight-for-age, height-for-age, and CD4 percent were not significant when included with the corresponding time-updated variables (data not shown).

**Table 2:** Effects of pre-antiretroviral therapy (ART) and time-updated characteristics on the risk of developing tuberculosis (TB) after ART initiation

	HR (unadjusted) 95 % CI	*P*	aHR (adjusted) 95 % CI	*P*
Age (at ART initiation)				
< 3 years	1.0		1.0	
≥ 3 years	0.63 (0.40–1.02)	0.06	0.54 (0.31–0.96)	0.04
Sex				
Male	1.0		1.0	
Female	1.51 (0.94–2.45)	0.09	1.88 (1.15–3.06)	0.01
Height-for-age Z score				
(time-updated)	0.65 (0.55–0.78)	<0.001	0.93 (0.73–1.20)	0.61
Weight-for-age Z score				
(time-updated)	0.62 (0.54–0.71)	<0.001	0.70 (0.56–0.86)	0.001
WHO stage (pre-ART)				
1 or 2	1.0		1.0	
3 or 4	1.82 (1.05–3.16)	0.03	1.32 (0.72–2.40)	0.37
CD4 %				
(time-updated)	0.94 (0.92–0.97)	<0.001	0.94 (0.91–0.97)	<0.001
Initial ART				
Included NVP	1.0		1.0	
Did not include NVP	0.51 (0.29–0.92)	0.02	0.95 (0.47–1.90)	0.87
Randomization				
A (3TC/ABC/NNRTI throughout)	1.0		1.0	
B (3TC/ABC/NNRTI throughout, ZDV until week 36)	0.75 (0.44–1.29)	0.31	0.83 (0.48–1.43)	0.50
C (3TC/ABC/ZDV throughout, NNRTI until week 36)	0.48 (0.26–0.89)	0.02	0.43 (0.22–0.81)	0.01

Note: final model includes all factors with *P* <0.1 in univariable analyses (Table 1)

*3TC* Lamivudine, *ABC* Abacavir, *aHR* Adjusted hazard ratio, *HR* Hazard ratio, *NNRTI* Non-nucleoside reverse transcriptase inhibitor, *NVP* Nevirapine, *ZDV* Zidovudine

Although a nevirapine-containing first-line ART regimen was univariably associated with risk of TB, this was no longer significant when age and current weight were included in the model, likely due to confounding, as all children under 3 years started nevirapine. However, a reduced risk of TB remained for those randomized to ART regimen C (induction-maintenance strategy) compared to regimen A (3-drug NNRTI-based regimen throughout) (*P* = 0.01). To investigate this further we restricted the analysis to TB incidence within 12 weeks of starting ART (with appropriate censoring) when Arms B and C were the same (i.e. a 4-drug ART regimen). Here we found no evidence of a difference for Arm B versus A (*P* = 0.6), with some limited evidence of a difference for Arm C versus A (*P* = 0.06), suggesting that this could be a chance effect.

### TB-associated immune reconstitution inflammatory syndrome (TB-IRIS)

Twenty-two TB cases (32 %) were reported as likely due to TB-IRIS, with a median time to diagnosis of 20 weeks following ART initiation (IQR, 4–42) versus 100 (24–151) in non-IRIS cases. Of the IRIS cases, 17 (77 %) were presumptive TB, and notably 17 (77 %) occurred in females.

Censoring non-IRIS cases in the same multivariable Cox model to estimate the impact of factors on the cause-specific hazard of developing TB-IRIS before TB non-IRIS, showed broadly similar results to Table 2, although power was lower with small numbers of events; the only significant associations were with weight and CD4 percent (data not shown). Censoring TB-IRIS cases in the same multivariable Cox regression analysis to estimate the impact of factors on the cause-specific hazard of developing TB non-IRIS before TB-IRIS, showed similar results in terms of identifying risk factors, except that sex was no longer significant: males versus females (HR = 1.28; 95 % CI, 0.72–2.29; *P* = 0.39).

### Outcomes

By the end of follow-up, of the 69 adjudicated TB cases, 3 (4 %) children had died compared to 43 (5 %) of those without TB (*P* = 0.81), and 3 (4 %) had a further diagnosis of recurrent TB in follow-up (occurring 11, 17, and 24 months after the first diagnosis).

### Co-trimoxazole analysis

After 96 weeks on ART, 760 children were eligible for the co-trimoxazole randomization; of these, 138 had a previous history of TB and were excluded, leaving 622 children in the analysis (Fig. 1b); 310 (50 %) children were randomized to continue co-trimoxazole and 312 (50 %) to stop, and baseline characteristics of children were well balanced by randomized group (Additional file 2: Table S2). Overall, 51 % were female and median age was 4 years. The median weight-for-age and height-for-age scores were −1.3 and −1.9, respectively, and CD4 percent was 32 %.

There were a total of 22 TB diagnoses over a median of 2 years follow-up after co-trimoxazole randomization. Two TB diagnoses were adjudicated as not TB by the ERC leaving 20 incident TB cases and 602 children with no TB at the end of follow-up. Of 20 adjudicated TB cases, 5 were definitive (25 %) and 15 (75 %) were presumptive.

**Fig. 3:**
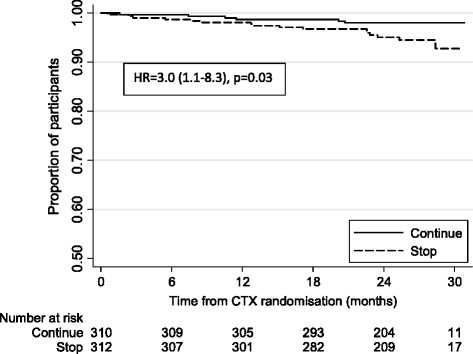
Time to tuberculosis diagnosis by co-trimoxazole randomization (after 96 weeks on antiretroviral therapy)

Time to TB diagnosis by randomized group is shown in Fig. 3. There were 5 adjudicated TB cases in the continue arm and 15 in the stop arm (HR = 3.0; 95 % CI, 1.1–8.3; *P* = 0.028), comparing stopping to continuing co-trimoxazole. Of the 5 cases in the continue arm, 1 was definitive and 4 were presumptive; of the 15 cases in the stop arm, 4 were definitive and 11 were presumptive. Adjusting for risk factors identified from the main analysis (including age, sex, CD4 percent, weight and current ART regimen) gave similar results (HR = 2.7; 95 % CI, 1.0–7.6; *P* = 0.055). Table 3 shows the characteristics of the children at the time of co-trimoxazole randomization by future TB diagnosis. On multivariable adjustment, along with stopping co-trimoxazole, only a lower current CD4 percent (*P* <0.001) was independently associated with a higher risk of TB. Repeating the analysis including children (*n* = 138) who had a history of TB gave similar results (data not shown).

**Table 3:** Characteristics of children who did and did not develop tuberculosis (TB) following co-trimoxazole randomization

At co-trimoxazole randomization	TB	No TB	Total	*P* value
	20 (3 %)	602 (97 %)	622	
Randomization				
Stop co-trimoxazole	15 (5)	297 (95)	312	
Continue co-trimoxazole	5 (2)	305 (98)	310	0.024
Age (at co-trimoxazole randomization)				
Median, IQR	6.5 (3.5–11.5)	7 (4–10)		
<5 years	10 (3)	280 (97)	290	0.75
Sex				
Male	8 (3)	295 (97)	303	
Female	12 (4)	307 (96)	319	0.43
CD4 median IQR (time-updated)				
CD4 %	18 (11.5–31)	33 (26–38)		<0.001
Weight median IQR (time-updated)				
Weight-for-age Z score	−1.0 (−2.1 to –0.5)	−1.3 (1.9 to –0.6)		0.60
Height median IQR (time-updated)				
Height-for-age Z score	−1.8 (−3.0 to –0.9)	−1.9 (−2.7 to –1.1)		0.92
WHO stage (pre-ART)				
3 and 4	13 (3)	378 (97)	391	0.84
ART randomization				
A (3TC/ABC/NNRTI throughout)	8 (4)	192 (96)	200	
B (3TC/ABC/NNRTI post week 36)	8 (4)	210 (96)	218	
C (3TC/ABC/ZDV post week 36)	4 (2)	200 (98)	204	0.46
ART at co-trimoxazole randomization				
3TC ABC EFV	4 (3)	134 (97)	138	
3TC ABC NVP	11 (4)	245 (96)	256	
ZDV 3TC ABC	4 (2)	196 (98)	200	
Other	1 (4)	27 (96)	28	0.58

Values are n (row%) unless otherwise stated

*3TC* Lamivudine, *ABC* Abacavir, *ART* Antiretroviral therapy, *EFV* Efavirenz, *IQR* Interquartile range, *NNRTI* Non-nucleoside reverse transcriptase inhibitor, *NVP* Nevirapine, *ZDV* Zidovudine

## Discussion

TB remains one of the major co-infections in HIV-infected children, but there are scarce prospective data on TB incidence in children starting ART. We therefore undertook an analysis of incident TB using prospectively collected data from the ARROW trial in Uganda and Zimbabwe, which is the largest pediatric trial to date of children initiating ART in Africa. We show here that TB incidence is much higher in HIV-infected children than in the general population in these countries, and that the risk is particularly high in the first 3 months of treatment. Strikingly, despite good immune reconstitution after 2 years of ART, continuation of co-trimoxazole led to significant reductions in incident TB, suggesting an additional role for co-trimoxazole prophylaxis in settings of high TB burden.

Available data on TB incidence in children on ART are mostly limited to retrospective analyses and observational cohorts [4–8, 23], with the inherent problems of missing data and loss to follow-up. The ARROW trial, which enrolled children in two countries with high HIV-TB burden, had a large sample size, high rates of follow-up over 4–5 years, and independently adjudicated clinical endpoints. We estimated an overall TB incidence rate of 1,900 per 100,000 child-years. This incidence is more than 40 times higher than recently reported rates in the Kampala district [24] and more than 50 times higher than notified rates of childhood TB in Zimbabwe [1]. In fact, the rates of TB in HIV-uninfected children would be even lower than in these reports as a substantial proportion of children included in these surveys were HIV-infected [24].

The incidence of TB disease varied over time following ART initiation and was highest in the first 3 months (8,800/100,000 child-years), as also observed in adults [25]. High TB rates in the first few months following ART initiation have been reported previously in children [4–8, 26]; contributory factors include undiagnosed prevalent TB, unmasking of TB as result of immune reconstitution (TB-IRIS), and ongoing risk due to pre-ART immune suppression and the required time for CD4 reconstitution. Compared to the first 3 months of ART, the rates of TB decreased 7-fold after 12 months, showing the benefits of ART in TB prevention. However, TB incidence after 1 year of ART still remained more than 25 times higher than that reported in pediatric populations in Uganda and Zimbabwe [1, 24]. These higher rates of TB on established ART may be due to higher exposure risk in HIV-infected households as well as to increased risk of relapse due to incomplete restoration of functional TB-specific immunity, despite numerical recovery of CD4 cells [27]. This is an important finding in the current era of universal ART recommendation for HIV-infected children, and suggests an additional benefit of early ART initiation, in line with recent trials in adults [9, 10].

The main predictors for developing TB were lower weight-for-age Z-score and lower CD4 percent. These are well-reported risk factors for overall mortality in HIV-infected children and highlight the importance of ART initiation prior to advanced disease as well as TB symptom screening prior to starting ART, particularly in malnourished and severely immunocompromised children. We also found some evidence that children younger than 3 years were at a higher risk, consistent with the natural history of TB, in which risk of progression from latent to active disease is greater at younger ages, likely due to less robust TB-specific immune responses [28, 29]. We found that female sex was associated with TB diagnosis, which seemed particularly driven by higher rates of TB-IRIS in girls. The association with sex is difficult to explain, and could be a chance finding given the relatively small number of TB diagnoses overall. However, there is an emerging literature on the influence of sex on outcome of infectious diseases from infancy onwards [30], which may be due to physiological or behavioral differences between sexes [31]. The sex bias may vary with age, indicating the influence of sex hormones on immune function at different stages of the life cycle. In adults, TB cases among men generally exceed those among women [32]. However, in children, a higher frequency of extra-pulmonary TB was reported among girls compared to boys [33].

We observed that children on a 4-drug induction-maintenance regimen including an NNRTI for the first 36 weeks were less likely to be diagnosed with TB compared to a standard 3-drug ART regimen. This may be a chance finding as we did not observe this same reduction in Arm B, where children were taking the same regimen up to week 36 (when restricting the analysis to TB cases within the first 12 weeks).

We showed that prolonged use of co-trimoxazole significantly reduced the risk of developing TB, despite high CD4 counts after 2 years of ART. Although there were relatively few events, the observed effect of co-trimoxazole on risk reduction was marked, with a 3-fold reduction in TB incidence among those continuing, compared to those stopping, co-trimoxazole. This is the first randomized evidence to support the epidemiological and in vitro observations regarding a potential prophylactic effect of co-trimoxazole on mycobacteria.

TB diagnosis in HIV-infected children is reputably challenging and TB over-diagnosis was possible; therefore, some reductions among children continuing co-trimoxazole may have been due to other respiratory infections diagnosed as TB. With no good current diagnostic methods for TB available in children [34], it will not be possible to increase the precision of childhood TB diagnosis in the near future. Therefore, it is important not only to prevent TB but also to prevent other respiratory infections that are frequently misdiagnosed as TB, to avoid prolonged anti-TB treatment. This may be particularly important for HIV-infected children who are frequently exposed to recurrent TB treatment [35] and who face numerous toxicities and drug-to-drug interactions between rifampicin and certain antiretrovirals.

The high risk of incident TB in this analysis, despite ART, highlights the importance of adjunctive strategies to reduce TB acquisition. Isoniazid preventative treatment at the start of ART has been recently shown to be more effective in reducing TB in HIV-infected adults in the TEMPRANO 12136 trial compared to ART alone [10]. Universal IPT recommendations for HIV-infected children were issued in 2012 [36], after the ARROW trial finished. In ARROW, only 36 (3 %) children reported taking isoniazid preventive treatment (IPT) and none of those on IPT developed TB*.* Whilst IPT is very effective in HIV-infected children who are exposed to TB [37], the role of pre-exposure IPT in children receiving ART has been debated. Madhi et al. [38] showed no benefit of isoniazid in children aged <1 year who were not exposed to TB. The lack of availability of single isoniazid formulations in developing countries, where isoniazid is mainly procured as co-formulated tablets with other anti-TB medications, has been a substantial barrier for implementation of IPT in HIV-infected adults and children [39].

This study had certain limitations. Most of the TB cases were presumptive because of the inherent challenges in confirming pediatric TB [40]. TB diagnosis is even more difficult in HIV-infected children as TB and HIV have overlapping clinical presentations, and children with HIV have frequent respiratory comorbidities. Consequently, TB in HIV-infected children in ARROW may have been under- or over-diagnosed. One strength of our study was that all reported TB events were reviewed by an independent ERC, and events that were adjudicated as unlikely due to TB were excluded or re-classified. The open-label design of the co-trimoxazole randomization could have led to bias in caregiver health-seeking behavior or clinician diagnoses; however, an effect of co-trimoxazole on TB prevention was not anticipated and adjudication of the events was undertaken blind to randomized group.

## Conclusions

In summary, this study showed that TB rates in HIV-infected children on ART are many-fold higher than those in the general pediatric population, particularly during the first 3 months of ART. These findings emphasize the importance of TB screening prior to ART initiation and the need to implement effective preventive treatment in HIV-infected children without active TB disease. Co-trimoxazole appears to reduce incident TB in HIV-infected children, even after several years of ART, and may contribute to preventing TB alongside the use of IPT. A co-formulated fixed dose combination of co-trimoxazole, isoniazid, and vitamin B6 has been recently developed for adults and older children and is being evaluated using this formulation in the REALITY trial [39, 41]. While a formulation still needs to be developed for younger children, this fixed dose combination could resolve unacceptably slow implementation of universal IPT and inadequate co-trimoxazole coverage in this population.

### Availability of data

The ARROW trial data are held at MRC CTU at UCL, which encourages optimal use of data by employing a controlled access approach to data sharing [42]. All requests for data are considered and can be initiated by contacting mrcctu.ctuenquiries@ucl.ac.uk.

## Additional files

Additional file 1: Table S1. Baseline characteristics of children by subsequent tuberculosis (TB) status including those with a history of TB. Values are n (row %) unless otherwise stated. (DOCX 24 kb) Additional file 2: Table S2. Characteristics of children by randomization to stop or continue co-trimoxazole after 96 weeks on antiretroviral therapy. Values are n (col %) unless otherwise stated. (DOCX 23 kb)
