# Supplementary material for: Tuberculosis incidence is high in HIV-infected African children but is reduced by co-trimoxazole and time on antiretroviral therapy
Source: BMC Med. 2016 Mar 23;14:50. doi: 10.1186/s12916-016-0593-7 (PMC4804479; doi:10.1186/s12916-016-0593-7)
Supplement: Additional file 1: Table S1. — Baseline characteristics of children by subsequent tuberculosis (TB) status including those with a history of TB. Values are n (row %) unless otherwise stated. (DOCX 24 kb) [file 12916_2016_593_MOESM1_ESM.docx]

**Table S1** Baseline characteristics of children by subsequent TB status including those with a history of TB

Values are n (row %) unless otherwise stated

|  | **TB** | **No TB** | **Total** | **p-val** |
| --- | --- | --- | --- | --- |
|  | **88 (7%)** | **1118 (93%)** | **1206** |  |
| **History of TB** | 19 (7) | 218 (93) | 237 | 0.64 |
| **Age (at baseline)** |  |  |  |  |
| Median, IQR (years) | 6 (2-11) | 6 (2-9) |  |  |
| < 3 years | 33 (9) | 337 (91) | 370 | 0.15 |
|  |  |  |  |  |
| **Sex** |  |  |  |  |
| Male | 36 (6) | 560 (94) | 596 | 0.10 |
| Female | 52 (9) | 558 (91) | 610 |  |
| **Centre** |  |  |  |  |
| Entebbe | 17 (9) | 171 (91) | 179 | 0.25 |
| JCRC | 22 (7) | 296 (93) | 264 |  |
| Harare | 22 (6) | 378 (95) | 257 |  |
| PIDC | 27 (9) | 273 (91) | 269 |  |
| **Height (median IQR)** |  |  |  |  |
| Height-for-age Z score | -2.9 (-3.9,-1.9) | -2.4 (-3.3,-1.5) |  | <0.001 |
| **Weight (median IQR)** |  |  |  |  |
| Weight-for-age Z score | -3.1 (-4.4,-1.9) | -2.1 (-3.2,-1.2) |  | <0.001 |
| **WHO stage** |  |  |  |  |
| 3 or 4 | 68 (8) | 778 (92) | 846 | 0.13 |
| **CD4 (median, IQR)** |  |  |  |  |
| CD4 % | 9 (5-13) | 12 (7-18) |  | 0.001 |
| CD4 count† | 142 (43-281) | 253 (102-395) |  | 0.005 |
| **Initial ART** |  |  |  |  |
| 3TC ABC EFV | 7 (5) | 136 (95) | 143 | 0.03 |
| 3TC ABC NVP | 29 (11) | 225 (89) | 254 |  |
| ZDV 3TC ABC EFV | 17 (6) | 288 (94) | 305 |  |
| ZDV 3TC ABC NVP | 35 (7) | 469 (93) | 504 |  |
| **Randomisation** |  |  |  |  |
| A (3TC/ABC/NNRTI throughout) | 36 (9) | 361 (91) | 397 |  |
| B (3TC/ABC/NNRTI throughout,  ZDV until week 36) | 31 (8) | 373 (92) | 404 |  |
| C (3TC/ABC/ZDV throughout,  NNRTI until week 36) | 21 (5) | 384 (95) | 405 | 0.10 |
| **Randomisation** |  |  |  |  |
| Clinical monitoring | 46 (8) | 560 (92) | 606 |  |
| Laboratory monitoring | 42 (7) | 558 (93) | 600 | 0.69 |

†in those over 5 years
