# Supplementary material for: Tuberculosis incidence is high in HIV-infected African children but is reduced by co-trimoxazole and time on antiretroviral therapy
Source: BMC Med. 2016 Mar 23;14:50. doi: 10.1186/s12916-016-0593-7 (PMC4804479; doi:10.1186/s12916-016-0593-7)
Supplement: Additional file 2: Table S2. — Characteristics of children by randomization to stop or continue co-trimoxazole after 96 weeks on antiretroviral therapy. Values are n (col %) unless otherwise stated. (DOCX 23 kb) [file 12916_2016_593_MOESM2_ESM.docx]

**Table S2** Characteristics of children by randomisation to stop or continue co-trimoxazole after 96 weeks on ART

Values are n (col %) unless otherwise stated

| At co-trimoxazole randomisation | **Continue**  **n=310** | **Stop**  **n=312** | **Total** |
| --- | --- | --- | --- |
| **Age** |  |  |  |
| Median, IQR | 4 (6.5-10) | 4 (7-10) |  |
| < 5 years† | 154 (50) | 136 (44) | 290 |
| **Sex** |  |  |  |
| Male | 154 (50) | 149 (48) | 303 |
| Female | 156 (50) | 163 (52) | 319 |
| **Centre** |  |  |  |
| Entebbe | 72 (23) | 76 (24) | 148 |
| JCRC | 71 (23) | 70 (22) | 141 |
| Harare | 59 (19) | 57 (18) | 116 |
| PIDC | 108 (35) | 109 (35) | 217 |
| **CD4 median (IQR)** |  |  |  |
| CD4 % | 33 (26-38) | 32 (26-38) |  |
| **Height median (IQR)**  Height-for-age Z score | -1.9 (-2.7,-1.1) | -1.9 (-2.6,-1.1) |  |
| **Weight median (IQR)**  Weight for age Z score | -1.2 (-1.9, -0.5) | -1.3 (-2.0,-0.6) |  |
| **WHO stage (pre-ART)**  1 or 2  3 or 4 | 122 (39)  188 (61) | 109 (35)  203 (65) | 231  391 |
| **ART randomisation** |  |  |  |
| A (3TC/ABC/NNRTI throughout) | 99 (32) | 101 (32) | 200 |
| B (3TC/ABC/NNRTI throughout,  ZDV until week 36) | 112 (36) | 106 (34) | 218 |
| C (3TC/ABC/ZDV throughout,  NNRTI until week 36) | 99 (32) | 105 (34) | 204 |
| **ART at co-trimoxazole randomisation** |  |  |  |
| 3TC ABC EFV | 63 (20) | 75 (24) | 138 |
| 3TC ABC NVP | 138 (45) | 118 (38) | 256 |
| ZDV 3TC ABC | 97 (32) | 103 (33) | 200 |
| Other | 12 (4) | 16 (5) | 28 |

†Children had to be older than 3 years to be eligible for randomisation so less than 5 years used as the cut off here.
